# Human T-cell leukemia virus type 1: oncogenic potential and vaccine development strategies

**DOI:** 10.3389/fcimb.2025.1587802

**Published:** 2025-08-15

**Authors:** Jorge Vasconez-Gonzalez, Isaac A. Suárez-Sangucho, Esteban Acosta-Muñoz, Luis Paz y Miño, Domenic Borja-Mendoza, John Altamirano Alexander-Castillo, Julia Saa, Natasha Salazar-Calvopiña, Paúl Cárdenas, Andrés López-Cortés, Esteban Ortiz-Prado

**Affiliations:** ^1^ One Health Research Group, Faculty of Health Science, Universidad de Las Americas, Quito, Ecuador; ^2^ Institute of Microbiology, Universidad San Francisco de Quito, Quito, Ecuador; ^3^ Cancer Research Group (CRG), Faculty of Medicine, Universidad de Las Américas, Quito, Ecuador

**Keywords:** HTLV-1, human T-cell leukemia virus type 1, oncogenesis, viral oncogenes, immune evasion, retrovirus, cancer

## Abstract

The human T-cell lymphotropic virus type 1 (HTLV-1) is a highly oncogenic retrovirus recognized as the causative agent of adult T-cell leukemia/lymphoma (ATLL) and HTLV-1-associated myelopathy/tropical spastic paraparesis (HAM/TSP). Among the key risk factors for ATLL development are high proviral load, reduced anti-Tax immune responses, and elevated levels of soluble interleukin-2 receptor. Unlike classical oncogenic viruses, HTLV-1 does not encode proto-oncogenes but instead drives cellular transformation through a combination of mechanisms, including viral gene dysregulation, chromatin remodeling, epigenetic reprogramming, persistent clonal expansion, immune evasion, and RNA-based modifications. Despite growing understanding of these molecular pathways, an effective prophylactic vaccine against HTLV-1 remains unavailable. However, several vaccine strategies including viral vector platforms, mRNA-based candidates, peptide vaccines, and dendritic cell-based approaches have shown promise in preclinical models. In this review, we provide a comprehensive synthesis of current knowledge on HTLV-1 oncogenesis, highlight the roles of viral proteins such as Tax and HBZ in immune evasion, and critically examine the state of vaccine development efforts aimed at controlling this neglected human retrovirus.

## 1 Introduction

Human T-lymphotropic viruses (HTLVs) constitute a family of human retroviruses classified as oncoviruses due to their ability to induce malignant transformation. To date, four distinct types have been identified: HTLV-1, HTLV-2, HTLV-3, and HTLV-4 (Eusebio-Ponce et al., 2019; Bryan and Tadi, 2025). Among them, HTLV-1 is the most clinically significant and was the first human retrovirus to be discovered. It was isolated in 1980 from T-cell lines derived from patients with cutaneous T-cell lymphoma and adult T-cell leukemia (Eusebio-Ponce et al., 2019; Forlani et al., 2021). Notably, it was also the first retrovirus conclusively linked to human disease (Coffin, 2015).

HTLV-1 is associated with several diseases, most notably adult T-cell leukemia/lymphoma (ATLL) and HTLV-1-associated myelopathy/tropical spastic paraparesis (HAM/TSP) (Kannian and Green, 2010). Although it can infect a variety of immune and endothelial cell types including T cells, B cells, monocytes, dendritic cells, and endothelial cells its transforming capacity is restricted to primary T lymphocytes, which play a central role in initiating and sustaining the adaptive immune response (Macatonia et al., 1992; Hanon et al., 2000; Forlani et al., 2021).

HTLV-1 is transmitted through parenteral, sexual, and vertical routes. Effective transmission notably requires direct transfer of infected cells, as the cell-free virus is largely non-infectious due to host cellular barriers that hinder viral propagation (Giam and Semmes, 2016; Eusebio-Ponce et al., 2019). In fact, free HTLV-1 virions exhibit low infectivity for most cell types. Following transmission, the majority of infected individuals remain asymptomatic throughout life. However, approximately 3%–5% of cases may progress to ATLL, a highly aggressive hematologic malignancy (Osame et al., 1986; Giam and Semmes, 2016).

## 2 HTLV-1 structure

HTLVs are enveloped retroviruses with an approximate diameter of 80 to 100 nm. HTLV-1, the most clinically relevant member of this group, contains a single-stranded RNA genome enclosed within an icosahedral capsid. This capsid is surrounded by a host-derived proteolipid bilayer that incorporates viral surface glycoproteins essential for cell entry (Verdonck et al., 2007; IARC Working Group on the Evaluation of Carcinogenic Risks to Humans, 2012; Bryan and Tadi, 2025).

Unlike other retroviruses, HTLV-1 particles are often polymorphic, which may partly explain their limited infectivity in the cell-free state (Meissner et al., 2017). The capsid protein (CA) consists of N- and C-terminal domains connected by a flexible linker and shows distinct structural characteristics compared with HIV-1 and equine infectious anemia virus (EIAV), including a dependence on the oxidation state of cysteine residues that may influence oligomerization and capsid assembly (Khorasanizadeh et al., 1999). Internally, HTLV-1 encodes a classic retroviral genomic structure, including long terminal repeat (LTR) sequences and three structural genes: *gag*, *pol*, and *env*. In addition, the pX region, contains regulatory genes (*tax*, *rex*) and accessory genes (*p12*, *p13*, *p30*, and *HBZ*) (IARC Working Group on the Evaluation of Carcinogenic Risks to Humans, 2012; Bryan and Tadi, 2025). This organization was first elucidated through complete sequencing of the integrated provirus in leukemic cells (Seiki et al., 1983; Haseltine et al., 1985).

**Figure 1:**
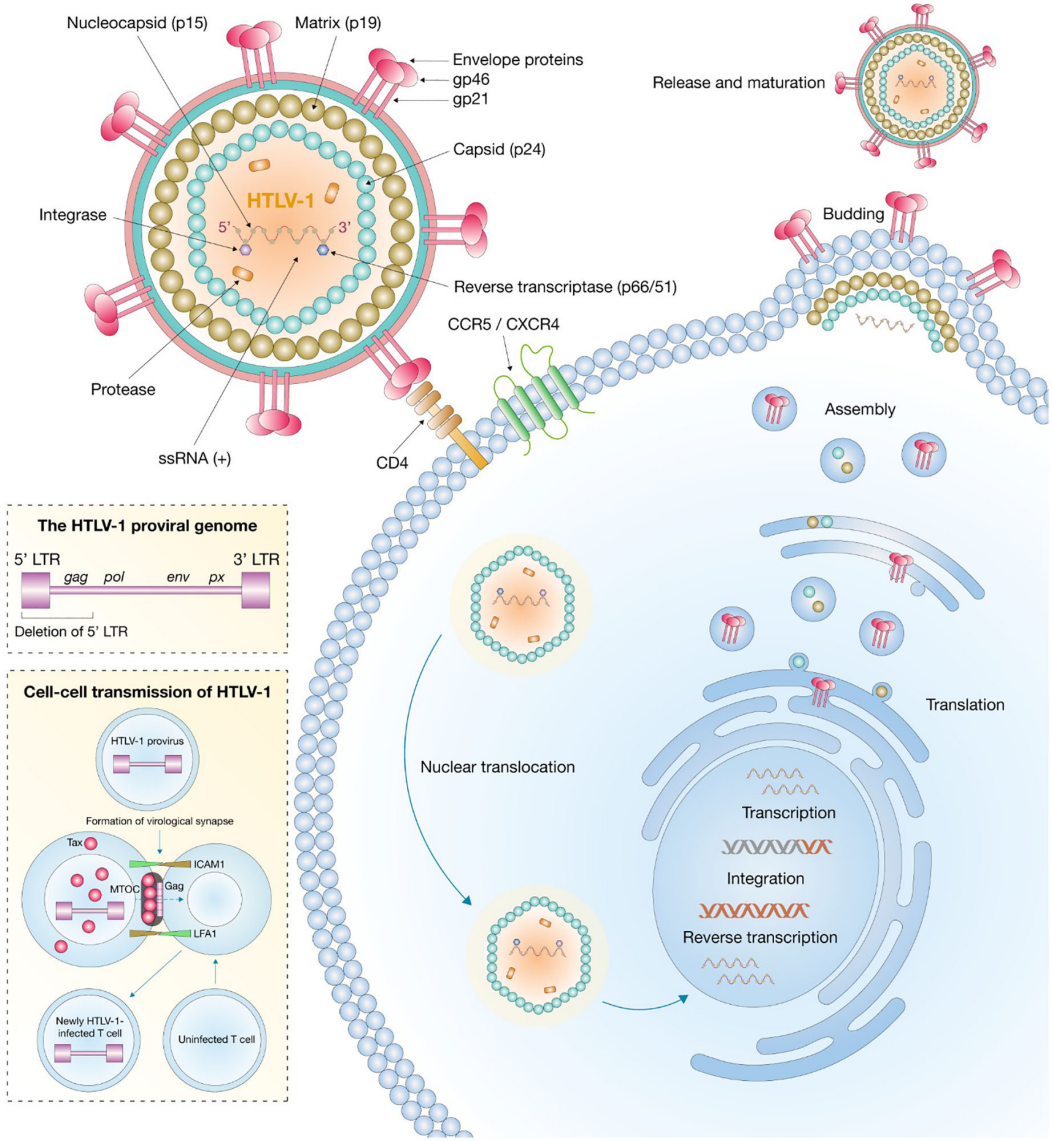
Structure and replication of HTLV-1.

The Gag precursor is cleaved by viral protease into p19 (matrix), p24 (capsid), and p15 (nucleocapsid), whereas the Env precursor undergoes cellular protease-mediated cleavage into gp46 (surface glycoprotein) and gp21 (transmembrane protein) (**Figure 1**) (IARC Working Group on the Evaluation of Carcinogenic Risks to Humans, 2012; Bryan and Tadi, 2025). These proteins are critical for viral assembly, infectivity, and cell-to-cell transmission, which occurs predominantly through virological synapses rather than free virion dissemination (Lairmore et al., 2011). The Tax protein (40 kDa) is a potent transactivator of viral and cellular genes involved in transformation and proliferation, whereas Rex (27 kDa) regulates the nuclear export and stability of viral RNAs (Gaudray et al., 2002; Lambert, 2009). The HBZ protein, transcribed from the antisense strand, plays key roles in immune evasion (Uchiyama, 1997), cell survival, and oncogenesis (Romanelli et al., 2013).

## 3 Epidemiology

It is estimated that between 5 and 10 million people are currently infected with HTLV-1 worldwide, although the true burden is likely underestimated due to limited screening in endemic regions (Gessain et al., 2023; Mendoza et al., 2024). Approximately 50% of these infections occur in equatorial Africa, where prevalence varies markedly between regions (Mendoza et al., 2024). In adult populations, seroprevalence ranges from 0.3% to 3%, with significantly higher rates in older individuals (Gessain et al., 2023). In some African communities, particularly among older women, prevalence can reach 10%–25% (Gessain et al., 2023). The geographic distribution of HTLV-1 is highly heterogeneous. While Eastern and Northern Africa exhibit lower prevalence rates, other regions—including the southwest of Japan, parts of South America, the Caribbean, Australo-Melanesia, and focal areas in the Middle East—are considered endemic zones (Tsukasaki and Tobinai, 2013; Eusebio-Ponce et al., 2019). In Indigenous Australian populations, HTLV-1 infection has reached hyperendemic levels, with reported seroprevalence as high as 33.6%, particularly among older men (Einsiedel et al., 2016). In Europe, data remain limited; however, in England and Wales, it was estimated that 36,300 individuals were living with HTLV-1 as of 2021 (Mendoza et al., 2024). Prevalence in Israel has also shown geographic variability, with higher rates among donors from Eastern Europe, Latin America, and the Middle East (Stienlauf et al., 2009). Major routes of transmission include breastfeeding, sexual contact, and exposure to infected blood. The risk of transmission is heightened in settings where public health infrastructure does not support routine screening or educational interventions (Radygina and Mochalova, 2024).

## 4 Transmission mechanisms of HTLV-1

### 4.1 Mother-to-child transmission of HTLV-1

Mother-to-child transmission represents a significant route for the dissemination of HTLV-1, accounting for approximately 15% to 25% of all infections (Bittencourt, 1998; Martin-Latil et al., 2012). The main way that vertical transmission occurs is through extended breastfeeding, particularly when it continues beyond 6 months. The risk of transmission in these cases can range from 4% to 31%, depending on how long and how often the infant is breastfed (Ngoma et al., 2019; Miyazawa et al., 2021; Vesterbacka et al., 2024). Various factors related to both the mother and the child can affect how likely this transmission is to happen. One of the most important factors is the level of proviral load in the mother’s breast milk—higher levels are strongly linked to a greater chance of the infant becoming infected (Hisada et al., 2002; Van Tienen et al., 2012). While it is less common, transmission can also happen during pregnancy or childbirth, with estimates suggesting this occurs in approximately 0.5% to 7% of cases (Denis et al., 1992; Berteau et al., 1994).

### 4.2 Sexual transmission of HTLV-1

Sexual transmission is one of the main ways adults acquire HTLV-1, especially in areas where the virus is not common (Paiva and Casseb, 2014; Martel and Gotuzzo, 2022). The virus is transmitted more efficiently from men to women than the other way around, which is likely because seminal fluid tends to carry a higher proviral load than cervicovaginal secretions (Murphy et al., 1989; Ayerdi et al., 2023). According to epidemiological studies, the estimated transmission rate among heterosexual couples is around 0.6 cases per 100 person-years (Roucoux et al., 2005). Risk factors that increase susceptibility include having multiple sexual partners, engaging in unprotected intercourse, the presence of genital ulcers, and a history of sexually transmitted infections (STIs), such as syphilis, which is associated with a 40-fold increase in HTLV-1 seropositivity (Kaplan et al., 1996; Martel and Gotuzzo, 2022). A longer duration of sexual relationships and higher viral loads further enhance the likelihood of transmission (Kaplan et al., 1996; Paiva and Casseb, 2014).

### 4.3 Transmission through blood and medical procedures

HTLV-1 is a bloodborne virus that is mainly spread through transfusions of contaminated blood products and through needle-sharing among people who inject drugs (Hewagama et al., 2014; Oliveira et al., 2018). This route of transmission is highly efficient because the virus is integrated into the DNA of infected lymphocytes, which are passed directly to the recipient (Cassar and Gessain, 2017; Kalinichenko et al., 2022). In addition to recreational drug use, medical procedures have also played a major role in the spread of HTLV-1 (Pépin et al., 2010). HTLV-1 can also be transmitted through solid organ transplants, which has led to calls for universal screening of both donors and recipients in regions where the virus is common (Osman, 2003; Yara et al., 2009). While less frequent, healthcare workers have occasionally been infected through occupational exposures such as needlestick injuries, highlighting the ongoing importance of strict biosafety measures (Weiss et al., 1985).

## 5 Oncological mechanisms of HTLV-1

### 5.1 Multifaceted oncogenic role of HTLV-1 Tax

Tax acts as a viral transactivator protein that plays a key role in regulating HTLV-1 gene expression. It forms a complex with pCREB, which then binds to viral DNA and recruits’ coactivators like CBP/p300 to help activate transcription. Tax also promotes the recruitment of positive transcription elongation factor b (P-TEFb), which supports the elongation phase of transcription (Zhou et al., 2006; Nyborg et al., 2010). In addition, Tax facilitates the assembly of transcriptional machinery at the viral long terminal repeat (LTR) by helping recruit TBP, general transcription factors, and RNA polymerase II. This is achieved through the formation of quaternary complexes that include histone-modifying enzymes, ultimately aiding in the production of viral RNA (de la Fuente and Kashanchi, 2004). Tax also enhances gene expression by increasing the binding of activating transcription factor 2 and CRE-binding protein to the 21-base pair repeat enhancer regions (Franklin et al., 1993). Furthermore, it interacts with BRG1, which helps bring in the core transcriptional machinery, leading to higher transcription activity and greater viral replication (Easley et al., 2010).

The stability of the Tax protein plays a key role in maintaining persistent HTLV-1 infection and contributing to its ability to cause cancer. Several mechanisms have been identified that help keep Tax stable within infected cells. For example, research has shown that Tax can interfere with the ubiquitin system, which not only prevents its own degradation but also activates NF-κB signaling by promoting the activity of ubiquitin kinases (Lavorgna and Harhaj, 2014). It was also found that heat shock protein 90 (HSP90) protects Tax from being broken down by the proteasome, helping to stabilize it and enhance NF-κB pathway activation (Gao and Harhaj, 2013). They also noted that when HSP90 is inhibited, Tax levels drop significantly in HTLV-1-transformed cells, highlighting the potential of HSP90 inhibitors as a possible therapeutic approach (Gao and Harhaj, 2013). Moreover, it has been shown that even the transient expression of Tax is sufficient to activate anti-apoptotic mechanisms, which remain active even when Tax expression decreases. Even low levels of Tax can activate NF-κB and induce IL-10 production, both of which play a crucial role in the leukemogenesis of ATLL. On the other hand, low-level Tax expression is sufficient for the survival of primary ATLL cells (Mahgoub et al., 2018; Hleihel et al., 2023). This evidence highlights Tax as a promising therapeutic target, as the absence of Tax renders primary ATLL cells unable to survive, despite the presence of somatic mutations and HBZ (Hleihel et al., 2023).

**Figure 2:**
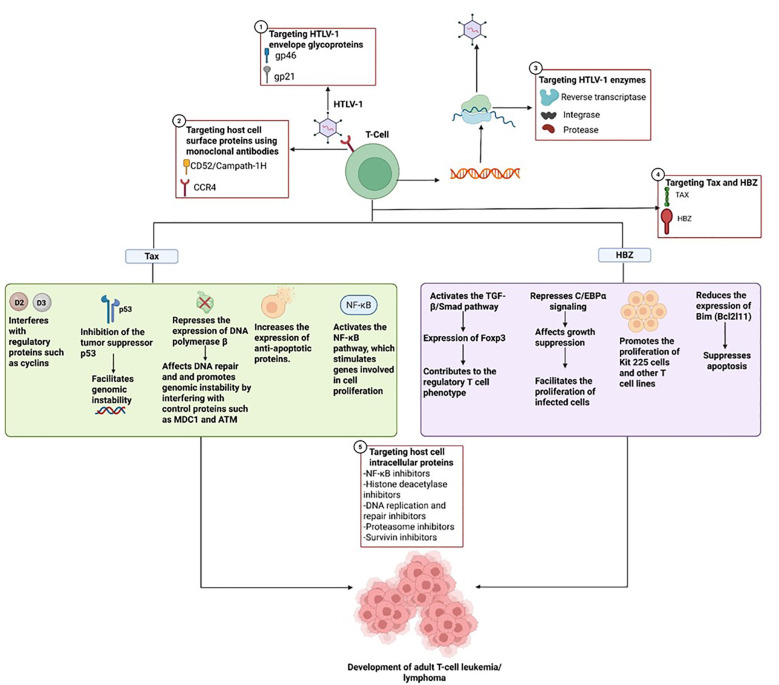
Oncogenic role of HTLV-1 mediated by Tax and HBZ, and possible therapeutic targets covering different stages ranging from T-cell infection by the virus, through the replication process, to mechanisms involved in oncogenesis.

Tax supports the proliferation of T cells by interfering with key regulatory proteins like cyclin D2 and D3 (**Figure 2**). This interference drives continuous, uncontrolled growth of mature T cells (Azran et al., 2004). Tax also suppresses the activity of the tumor-suppressor protein p53, disrupting normal cell cycle checkpoints and contributing to genomic instability an early step in the development of ATLL (Pise-Masison and Brady, 2005; Tabakin-Fix et al., 2006; Currer et al., 2012). Additionally, Tax can inhibit the transcription of human telomerase reverse transcriptase (hTERT), resulting in reduced telomerase activity in affected cells (Gabet et al., 2003). It also downregulates DNA polymerase β, a key enzyme in DNA repair processes, thereby impairing several repair pathways, including mismatch repair, base excision repair, and nucleotide excision repair (Azran et al., 2004). On top of that, Tax contributes to genomic instability by disrupting the function of checkpoint proteins like MDC1 and ATM (Mühleisen et al., 2014; Mohanty and Harhaj, 2020).

In addition, Tax increases the expression of anti-apoptotic proteins from the Bcl-2 family (Mühleisen et al., 2014; Mohanty and Harhaj, 2020) and upregulates XIAP through the activation of the NF-κB pathway (Kawakami et al., 1999). Tax-driven activation of NF-κB occurs through both the canonical and non-canonical signaling pathways. In this process, Tax binds to NEMO, which helps recruit the IKK complex and results in sustained NF-κB activation (Cheng et al., 2012; Mohanty and Harhaj, 2020). The IKK complex composed of IKKα, IKKβ, and NEMO triggers phosphorylation events of IκB and NF-Kb (Hinz and Scheidereit, 2014). More specifically, IKK phosphorylates IκB, marking it for ubiquitination and subsequent degradation, which allows NF-κB to move into the nucleus (Mohanty and Harhaj, 2020). A non-canonical NF-κB pathway also exists and is activated by signals such as B-cell activating factor, lymphotoxin-beta receptor, and CD40 (Mohanty and Harhaj, 2020).

Tax also promotes cell survival by linking IKK signaling to autophagy pathways, further contributing to cellular transformation (Cheng et al., 2012). Beyond NF-κB, it activates PI3K and AP-1, promotes trimethylation of histone H3K27, and engages EZH2, leading to gene silencing (Peloponese and Jeang, 2006; Fujikawa et al., 2016). Tax directly interacts with NF-κB subunits (p50, p52, RelA, c-Rel) and NF-κB precursors (p100, p105), enabling their activation and nuclear translocation (Li and Gaynor, 2018). Through these interactions, Tax enhances transcriptional activity and supports T-cell transformation (Mohanty and Harhaj, 2020).

Tax also activates TRAF6 and stabilizes MCL-1 via K63-linked polyubiquitination, conferring resistance to apoptosis (Mohanty and Harhaj, 2020). Ubiquitination at lysines K263, K280, and K284 regulates Tax stability and NF-κB activity through platforms that recruit IKK and TAK1 (Lavorgna and Harhaj, 2014). The K63-linked polyubiquitination of Tax enables the recruitment of kinases such as IKK and TAK1, facilitating the activation of NF-κB. Furthermore, Tax contributes to sustained NF-κB activation by interacting with both NEMO and Optineurin (Lavorgna and Harhaj, 2014). In response to DNA damage, Tax is monoubiquitinated at lysine residues K280 and K284, a modification that leads to its dissociation from nuclear bodies containing SC35. This change promotes the export of Tax from the nucleus through the CRM1-dependent pathway (Gatza et al., 2007; Lavorgna and Harhaj, 2014).

Tax can also be modified through SUMOylation, primarily at lysine residues K280 and K284. This modification plays a key role in regulating Tax’s movement from the cytoplasm into the nucleus, where it can interact with p300, RelA, and NEMO (Lavorgna and Harhaj, 2014; Mohanty and Harhaj, 2020). In addition, phosphorylation of Tax contributes to gene expression by activating the ATF/CREB and NF-κB pathways and promotes its accumulation in nuclear bodies activation (Mohanty and Harhaj, 2020). Mohanty et al. observed in their study that Tax can also be phosphorylated by VEGFR2. This phosphorylation protects Tax from degradation by autophagy and also stabilizes it. Therefore, drugs targeting VEGFR2 represent a promising therapeutic strategy, as its inhibition leads to apoptosis of infected T cells through Tax degradation. This, in turn, disrupts the NF-κB and JAK/STAT signaling pathways (Mohanty et al., 2024). Acetylation also plays a role when Tax is acetylated, it interacts with p300 to further support NF-κB activation (Mohanty and Harhaj, 2020).

Finally, Tax also contributes to immune evasion. It has been shown to inhibit interferon-β-inducing adaptors, disrupting their role as cellular sensors and reducing the production of type I interferons (Hyun et al., 2015). Tax also interferes with type I IFN signaling pathways mediated by RIG-I and MDA-5, making cells more vulnerable to viral infections (Hyun et al., 2015). Additionally, Tax has been found to interact with RIPK1, which affects the production of IFN-α, and with STING, thereby impairing the induction of IFN-β. These combined actions help create an immunosuppressive environment (Mohanty and Harhaj, 2023).

### 5.2 HBZ and its multifaceted role in oncogenesis

HBZ is a nuclear protein that consists of three main structural domains: an activation domain (AD) at the N-terminus, a central domain (CD), and a basic leucine zipper (bZIP) domain (Ma et al., 2017). Within the activation domain, two LXXLL-like motifs interact with the KIX domain of the transcriptional coactivators CBP/p300, which are crucial for chromatin remodeling and transcriptional regulation. These motifs are particularly important for HBZ’s ability to activate the TGF-β/Smad signaling pathway. This activation leads to the induction of Foxp3 expression, promoting a regulatory T cell phenotype (**Figure 2**) (Ma et al., 2016; Zhao, 2016).

HBZ has also been shown to antagonize Tax-mediated transcriptional activation through the 5′ LTR promoter, likely via interactions with CREB and CBP/p300 (Yoshida et al., 2008; Matsuoka and Green, 2009). Additionally, it can repress C/EBPα signaling, a pathway implicated in growth suppression by physically interacting with C/EBPα and impairing its DNA-binding capacity. This repression facilitates the proliferation of HTLV-1-infected cells, a key event in leukemogenesis (Zhao and Matsuoka, 2012; Zhao, 2016). HBZ also stimulates the expression of JunD and promotes the production of its alternative isoform, ΔJunD, through the nuclear retention of RPS25. This cooperation with JunD and Sp1 enhances transcription from the 3′ LTR and upregulates the expression of hTERT, thereby reinforcing proliferative and survival signaling (Zhao and Matsuoka, 2012; Gazon et al., 2018).

Apart from its role as a protein, HBZ mRNA itself plays a significant role in oncogenesis. It has been found to promote the proliferation of Kit 225 cells and other T-cell lines by forming stem-loop structures that activate E2F1 and its downstream targets, thereby encouraging progression from the G1 to the S phase of the cell cycle (Satou et al., 2006). In addition to promoting cell growth, HBZ RNA also helps cells evade apoptosis by downregulating Bim (*Bcl2l11*), a key pro-apoptotic gene. This occurs through its interference with FoxO3a, a transcription factor that normally activates *Bim* and *FasL*, by preventing its proper localization to the nucleus and its ability to bind DNA (Tanaka-Nakanishi et al., 2014).

HBZ also contributes to oncogenesis by upregulating oncogenic microRNAs (oncomiRs) at the posttranscriptional level. Higher levels of HBZ expression have been associated with the overexpression of specific oncomiRs in CD4^+^ T cells from individuals infected with HTLV-1, a pattern that supports the development of malignant features (Vernin et al., 2014; Mitagami et al., 2015).

Additionally, HBZ plays a significant role in immune evasion. It weakens CD4^+^ T-cell responses and disrupts interferon-gamma (IFN-γ) signaling by suppressing the canonical NF-κB and AP-1 pathways. These actions contribute to immune tolerance and enable the virus to persist in the host (Zhao, 2016). This protein has also been reported to stimulate the production of co-inhibitory molecules, enhance FOXP3 expression in coordination with TGF-β signaling, and increase IL-10 production, all of which contribute to the suppression of immune activation. Furthermore, by downregulating canonical NF-κB signaling, HBZ helps prevent the expression of cellular senescence markers in infected cells (Tan et al., 2022; Mohanty and Harhaj, 2023).

### 5.3 Additional proteins involved in oncogenesis

Rex is a 27-kDa phosphoprotein that functions as a posttranscriptional regulator, enhancing the expression of both spliced and unspliced viral mRNAs. It is also essential for maintaining the proper balance between these mRNAs, a process critical for the production of infectious viral particles (Ahmadi Ghezeldasht et al., 2013). The accessory protein p13 has been shown to induce an inward K^+^ current within mitochondria, which leads to an increase in the production of reactive oxygen species (ROS). In addition, p13 has been reported to enhance the effects of pro-apoptotic stimuli, including Fas ligand (FasL) and C2-ceramide (Silic-Benussi et al., 2010).

On the other hand, 12-kDa protein (p12I) is necessary for the infection of primary lymphocytes and plays an important role during the early stages of HTLV-1 infection. Additionally, by binding to the heavy chain of MHC I, p12I renders it susceptible to degradation and may reduce the presentation of viral peptide MHC I complexes on the surface of infected cells, thereby protecting them from lysis by cytotoxic T lymphocytes (Ahmadi Ghezeldasht et al., 2013).

The p30 protein inhibits DNA repair via homologous recombination by targeting the MRE11/RAD50/NBS1 complex and instead promotes the error-prone non-homologous end joining (NHEJ) DNA repair pathway. This shift facilitates the accumulation of mutations in the host genome and increases the cumulative risk of cellular transformation (Baydoun et al., 2011).

### 5.4 Role of the JAK/STAT, PI3K/AKT, and NF-κB signaling pathways

Signaling through hormone and cytokine receptors is the most widely accepted model for JAK activation; this process leads to the aggregation of JAK proteins within lipid rafts of the cell membrane (Bellon and Nicot, 2024). Once activated, this pathway results in a loss of phosphatase expression. Additionally, IL-2 has been shown to promote T-cell proliferation, whereas IL-13 exerts both proliferative and anti-apoptotic effects via the JAK/STAT pathway (Islam et al., 2021; Bellon and Nicot, 2024).

It has been reported that the p12 protein interacts with the interleukin-2 receptor β chain and the IL-2Rγ chains. This interaction activates the Janus kinase/signal transducer and activator of transcription 5 (JAK/STAT5) signaling pathway, which is involved in the infection of quiescent primary T cells and the formation of syncytia (Kannian and Green, 2010).

On the other hand, the PI3K/AKT signaling pathway has been identified as playing a role in cell survival, as well as in regulating cell cycle progression. Moreover, AKT activation leads to the inhibition of p53. Jeong et al. indicate that p53 siRNA contributes to cell survival by preventing LY294002-induced apoptosis (Jeong et al., 2008). In addition, Tax regulates the expression of Bcl-3 by activating the PI3K/AKT signaling pathway, a mechanism that has been linked to the increased proliferation of HTLV-1-infected T cells (Saito et al., 2010; Marino-Merlo et al., 2023).

The activation of the NF-κB pathway is essential for both the survival and proliferation of HTLV-1-infected T cells. Both the canonical and non-canonical branches of this pathway contribute to the upregulation of genes involved in cell proliferation, cell cycle progression, and anti-apoptotic functions (Harhaj and Giam, 2018; Zhao et al., 2022). However, sustained NF-κB activation also leads to the accumulation of R-loops nucleic acid structures that, when resolved, can result in DNA double-strand breaks (Giam and Pasupala, 2022). These breaks contribute to genomic instability, including insertions, deletions, and other structural alterations. Over time, such mutations may inactivate tumor-suppressor genes or disrupt regulatory feedback within the IKK/NF-κB pathway, promoting the development of adult ATLL (Giam and Pasupala, 2022).

## 6 HTLV-1 vaccine platforms

### 6.1 Viral vector vaccines

Viral vectors represent a relatively recent approach in vaccine development, using engineered viruses to deliver specific immunogens into the host. These vectors can trigger innate immune responses without requiring an adjuvant. Because they are capable of infecting host cells and expressing foreign antigens, they promote effective antigen presentation and activate major histocompatibility complex (MHC) pathways, ultimately eliciting a strong cellular immune response (McCann et al., 2022). This platform offers several advantages, including efficient expression of intracellular antigens, the induction of a potent cytotoxic T-cell response, and the stimulation of innate immunity resulting in the production of interferons and pro-inflammatory cytokines (Santana et al., 2023).

A variety of viral vectors have been employed to express HTLV-1 genes, including adenovirus (AdV), cytomegalovirus (CMV), vaccinia virus (VV), variola virus (VARV), and baculovirus (BV) (Letafati et al., 2024). One of the earliest attempts to develop a vaccine against HTLV-1 was carried out by Shida et al., who engineered recombinant vaccinia viruses to carry the HTLV-1 envelope gene. This gene was inserted into the hemagglutinin (HA) gene locus of the vaccinia virus, which served as a novel insertion site for foreign genetic material (Shida et al., 1987). By 1992, Ford et al. designed recombinant vaccinia viruses named RVV E1, RVV E2, and RVV E3 to express three different versions of the HTLV-1 envelope proteins (Ford et al., 1992) (**Table 1**).

**Table 1:** HTLV-1 viral vector vaccines.

Study subject	Result	Reference
Rabbits	A single inoculation was sufficient to induce the production of antibodies against Env proteins and to provide protection against infection.	(Shida et al., 1987).
Mice	Balb/c mice showed a poor response to immunization with the three RVV constructs.	(Ford et al., 1992).
Rabbits	Two inoculations were sufficient to confer protection against viral challenge 5 months after immunization. However, the combination of ALVAC-Env and two boosters did not provide protection.	(Franchini et al., 1995)
Monkeys	Following immunization, cytotoxic T lymphocyte activity against the Env protein was observed. Additionally, no antigens or provirus were detected in peripheral blood mononuclear cells.	(Ibuki et al., 1997)
Monkeys	Antibodies against the HTLV-1 Env protein were produced.	(Kazanji et al., 2001).
Japanese macaques	rVV reduced the proviral load and the number of cells expressing Tax. Additionally, it caused the T lymphocytes to have a much stronger response.	(Sugata et al., 2016).
Mice	CD4^+^ and CD8^+^ T lymphocytes developed polyfunctional dual-positive responses for TNF-α/IFN-γ and TNF-α/IL-2	(Daian E Silva et al., 2023).

In 1995, the full-length envelope protein of the HTLV-I (1711) virus was expressed using highly attenuated poxvirus vaccine vectors ALVAC and NYVAC. These vectors were used to immunize white rabbits; however, even after booster doses, no sustained immunity was observed, as the animals became infected with HTLV-1 after a few months (Franchini et al., 1995). Two years later, the recombinant vaccinia virus WR-SFB5env, which synthesizes the gp46 envelope protein, was developed. It was observed that WR-SFB5env expressed high levels of gp46 and was capable of inducing anti-HTLV-I Env antibodies, including neutralizing antibodies, which were maintained at high levels for up to 136 weeks (Ibuki et al., 1997). For their part, Kazanji et al. evaluated the immunogenicity and efficacy of two NYVAC-based vaccine candidates. They found that sensitizing the animals with a single dose of env DNA, followed by immunization with the NYVAC-HTLV-1 *gag* and *env* vaccine at months 6, 7, and 8, protected all three animals against exposure to HTLV-1-infected cells (Kazanji et al., 2001).

In another study involving vaccination with recombinant vaccinia virus (rVV) expressing either the Tax or HBZ protein, administered to rhesus monkeys and mice, it was observed that rVV expressing HBZ could induce HBZ-specific CD4+ and CD8+ T-cell responses. However, compared with the Tax protein, the immunogenicity of HBZ was low (Sugata et al., 2016). More recently, in 2023, a recombinant modified vaccinia Ankara virus (MVA-HBZ) and a plasmid DNA vector (pcDNA3.1(+)-HBZ) expressing a multiepitope protein based on HBZ peptides were developed. The results highlight the potential of the HBZ multiepitope protein, expressed from both the plasmid DNA and the poxviral vector, as candidates for a therapeutic vaccine (Daian E Silva et al., 2023).

### 6.2 Protein/peptide vaccines

Peptide vaccines are composed of the most immunogenic protein fragments and are capable of stimulating the humoral immune response through antibody production, thereby reducing the risk of adverse effects (Santana et al., 2023). The peptides present in this type of vaccine have the ability to stimulate antibody production, and when combined with adjuvants, they enhance immune responses. Their efficacy has been demonstrated in vaccine development trials (Letafati et al., 2024). Vaccination with peptides based on T- and B-cell epitopes, constructed by conjugating gp46 (aa 181–210) with a branched poly-lysine oligomer, was tested in rats and rabbits and demonstrated high levels of neutralizing antibodies against HTLV-1 (Seighali et al., 2023).

A study conducted in rabbits revealed that synthetic gp46 peptides containing amino acids 190–199 and 180–204 are effective in generating neutralizing antibody responses. Upon reinfection with HTLV-1, no detectable provirus was found in these vaccinated rabbits (Tanaka et al., 1994). The use of synthetic peptides is a promising strategy for the development of a vaccine targeting the envelope of this pathogen (Santana et al., 2023). The most recent vaccines are described in **Table 2**.

**Table 2:** HTLV-1 protein/peptide vaccines.

Peptide	Study subject	Result	Reference
Peptide construct named MVFMF2	Rabbits and mice	Protection against cell-associated viral infection was not achieved	(Frangione-Beebe et al., 2000)
Peptide composed of cytotoxic T lymphocyte epitopes restricted by HLA-A*0201.	Mice	Cellular responses against the epitopes were successfully induced	(Sundaram et al., 2003)
Peptides (WCCR2T y CCR2T)	Mice	High antibody titers were obtained that were able to prevent syncytium formation.	(Sundaram et al., 2004)
Peptides composed of one B cell epitope and three cytotoxic T lymphocyte epitopes restricted by HLA-A*0201	Monkeys	Antibodies and IFN-γ-producing cells against Env and Tri-Tax were generated	(Kazanji et al., 2006)
Chimeric particle of the hepatitis B virus core (HBc) incorporating the HTLV-1 Tax epitope restricted by HLA-A*0201	Mice	HTLV-1-specific CD8^+^ T cells were induced	(Kozako et al., 2009)
HTLV-1 Tax epitope restricted by HLA-A*0201 (OML/Tax)	Mice	OML/Tax generated a specific interferon-gamma response against HTLV-1	(Kozako et al., 2011)
Immunodominant epitopes of Tax, gp21, gp46, and Gag	Mice	A high titer of sIgA was successfully stimulated when the vaccine was administered intranasally	(Kabiri et al., 2018)
tTax-tEnv:mFcγ2a and tTax-tEnv: His,	Mice	tTax-tEnv:mFcγ2a induced an increase in IFN-γ production.	(Shafifar et al., 2022)

### 6.3 DNA vaccines

This type of vaccine is capable of generating adaptive immune responses comparable with those produced after administration of attenuated pathogen vaccines while maintaining the safety profile of subunit vaccines. Consequently, they are suitable for administration in immunocompromised individuals (Kozak and Hu, 2024). This type of vaccine is composed of a plasmid responsible for encoding an immunogen, which is administered into the body to generate a targeted immune response (Letafati et al., 2024).

One of the first studies of this type of vaccine against HTLV-1 was conducted in 1987, where the immunization of animals with products from the HTLV-I *env* gene produced in *Escherichia coli* was evaluated. The results revealed that animals immunized with the *env* product produced antibodies against HTLV-I gp68 and gp46, and their sera were found to strongly inhibit syncytium formation in a feline fibroblast cell line induced by HTLV-I (Nakamura et al., 1987). Later, in 1997, two plasmids encoding the full HTLV-1 envelope proteins were evaluated, with or without recombinant Baculovirus gp62 protein boosts, in BALB/c mice. The authors concluded that a single DNA inoculation expressing the HTLV-I *env* gene could stimulate memory B-cell clones capable of responding effectively to subsequent encounters with HTLV-I envelope proteins and a specific helper T-cell response in mice (Grange et al., 1997). By the year 2000, Armand et al. concluded, after evaluating two plasmids, that the choice of vectors would be crucial for the design of genetic vaccines against HTLV-I (Armand et al., 2000) (**Table 3**).

**Table 3:** HTLV-1 DNA vaccines.

Administered component	Study subject	Result	Reference
Products of the HTLV-I *env* gene produced in *Escherichia coli*.	Monkeys	Antibodies against HTLV-I gp68 and gp46 were produced.	(Nakamura et al., 1987)
Plasmids encoding the HTLV-I envelope proteins	Mice	Protein boosts induced a strong antibody response in previously sensitized mice	(Grange et al., 1997)
Recombinant Ad5-HTLV-I-env.	Rats	In rats sensitized and boosted with recombinant vaccinia virus carrying the HTLV-I *env* gene, antibody production against the gp21 and gp46 proteins was achieved.	(Kazanji et al., 1997)
Tax-coding DNA	Rats	Cytotoxic T lymphocyte (CTL) activity specific for Tax was produced	(Ohashi et al., 2000)
Two types of plasmids containing the complete coding sequence of the *env* gene	Mice	DesEnv generated a higher humoral response with improved neutralizing properties	(Armand et al., 2000)

### 6.4 Dendritic cell-based vaccines

Dendritic cell-based constructs have been proposed as therapeutic vaccines capable of inducing antigen-specific CD8+ T cell responses (Seighali et al., 2023; Letafati et al., 2024). Sagar et al. were among the first to suggest a dendritic cell-based vaccine candidate against HTLV-1, which utilized the Tax (11-19) epitope and effectively induced antigen-specific CD8+ T cells. Moreover, administration of Freund’s adjuvant reduced TGF-β levels and enhanced the CD8+ T cell response (Sagar et al., 2014) (**Table 4**).

**Table 4:** HTLV-1 dendritic cell-based vaccines.

Administered component	Study subject	Result	Reference
Tax(11-19) epitope	Mice	An antigen-specific CD8^+^ T cell response was generated.	(Sagar et al., 2014)
Autologous dendritic cells pulsed with Tax peptides corresponding to the cytotoxic T lymphocyte epitopes.	Humans	Cytotoxic T lymphocyte responses against Tax were generated, and one patient achieved complete remission.	(Suehiro et al., 2015)
Therapy with dendritic cells pulsed with HTLV-1 Tax(180–188).	Rats	Proviral load was successfully reduced, and Tax-specific CD8^+^ T lymphocytes were generated.	(Ando et al., 2017)

### 6.5 mRNA vaccines

mRNA vaccines have been shown to possess high potency, safety, and efficacy (Gote et al., 2023). These types of vaccines exert their protective effects by inoculating target cells with mRNA fragments related to a viral protein, enabling the cells to express this protein in order to trigger recognition by the immune system (Letafati et al., 2024).

In 2024, a codon-optimized mRNA encoding the HTLV-1 envelope (Env) was developed and evaluated. Results in animal models revealed that three rabbits were partially protected and three were fully protected against HTLV-1 exposure. It was also observed that in those immunized with Env mRNA-LNP, the proviral load and viral gene expression were significantly lower, in addition to showing an increase in CD4+/IFN-γ+ and CD8+/IFN-γ+ T cells (Tu et al., 2024).

### 6.6 HTLV-1 vaccine challenges

Among the main challenges in developing an effective vaccine against HTLV-1 are the virus’s ability to infect and persist in CD4^+^ T cells, as well as its capacity for cell-to-cell transmission, which facilitates immune evasion (Letafati et al., 2024). Furthermore, understanding the mechanisms by which the immune response can control HTLV-1 infection is necessary for vaccine design. However, with this type of virus, there is an additional challenge due to the limited knowledge about its protective mechanisms and pathogenesis (Santana et al., 2023). It is essential to thoroughly understand the gp46 subunit and its roles and configuration, as it may contribute to the development of successful vaccines. Therefore, the development of tools for its research and understanding is crucial (Letafati et al., 2024).

## 7 New therapies

### 7.1 Mogamulizumab

Is a humanized monoclonal antibody against CCR4 (**Figure 2**) that leads to antibody-dependent elimination of CCR4+ cells. It is used for the treatment of T-cell lymphomas (Meyerowitz et al., 2023). In a multicenter, randomized, phase 3 study in which 34 and 33 patients were randomized to the mogamulizumab and placebo arms, respectively, the results revealed that the mogamulizumab arm showed a significant reduction in HTLV-1 proviral load (−59.39% ± 29.91% vs. placebo 2.32% ± 36.31%) (Sato et al., 2024).

On the other hand, in an uncontrolled phase 1–2a study involving patients with HAM/TSP, it was observed that by day 15, the proviral load was reduced by 64.9%. By day 29, the number of HTLV-1 proviral copies per milliliter of cerebrospinal fluid (CSF) decreased by 41.4% (Sato et al., 2018). Additionally, in a study evaluating long-term safety and efficacy, the results revealed that 19% of participants developed neutralizing antibodies, and after 4 years, the proviral load decreased by 60.7% in peripheral blood and 66.3% CSF (Sato et al., 2023).

### 7.2 EZH2 inhibitors

Koseki et al. reported in their study that EZH2 and EZH1/2 inhibitors are capable of reducing proviral loads and increasing IL-10 levels. They also trigger the inhibition of proliferation in infected cells and enhance early apoptosis, as indicated by annexin-V(+) 7-aminoactinomycin D(−) staining (Koseki et al., 2023). It has been described that it is possible to reverse the epigenetic alteration and even eliminate leukemic cells infected with the virus through pharmacological inhibition of EZH2. Moreover, by inhibiting both EZH2 and DZNep, apoptosis of ATLL cells can be induced (Fujikawa et al., 2016; Letafati et al., 2025).

### 7.3 Pomalidomide

In T-lymphocyte cell lines immortalized and infected with HTLV-1, pomalidomide increases the expression of MHC-I, ICAM-1, and B7-2/CD86 and also enhances susceptibility to cytotoxicity mediated by NK cells (Davis et al., 2019). In a Rhesus macaque model, Gutowska et al. observed that pomalidomide induced immune activation, marked by increased proliferation of CD4^+^, CD8^+^, and NK cells. It was also reported that the specific humoral response against the virus was stronger, and there was an increase in the levels of antibodies directed against viral antigens (Gutowska et al., 2022).

### 7.4 Alcoholic extract from *Eucalyptus camaldulensis*

Abu-Jafar et al., in their study, demonstrated that the tannin extract of *Eucalyptus camaldulensis* inhibited the activation of NF-κB, SRF-dependent promoters, and the HTLV-1 LTR induced by Tax, and was even capable of preventing the degradation of IκBα (Abu-Jafar et al., 2020).

### 7.5 (E)-3-Phenyl-5-(phenylamino)-2-styryl-1,3,4-thiadiazol-3-ium chloride derivatives

Sousa-Pereira et al. reported IC_50_ values for all compounds in the range of 1.51–7.70 μM in both HTLV-1-infected and uninfected cells. These compounds may induce necrosis after 24 h in Jurkat and MT2 cell lines (Sousa-Pereira et al., 2020).

## 8 Conclusion

Although this virus was the first retrovirus identified as a causal agent of diseases in humans, it is often overlooked and, at times, forgotten, even becoming an unattended public health problem. The cellular and molecular processes through which HTLV-1 leads to the development of ATLL are complex, as they involve a series of mechanisms such as genomic integration, epigenetic reprogramming, persistent clonal expansion, and RNA-based modifications and alternative splicing, processes in which various proteins, including Tax and HBZ, play a role. Understanding the oncogenic mechanisms of this virus is crucial for developing therapies to prevent cancer associated with this infection and for developing effective vaccines. Various platforms have been studied to create a compelling and safe vaccine in recent years, but a safe vaccine that can prevent HTLV-1 infection has not yet been produced. It is also important to emphasize the limited number of clinical trials published regarding their development.
